# Advances in medical polyesters for vascular tissue engineering

**DOI:** 10.1186/s11671-024-04073-x

**Published:** 2024-08-08

**Authors:** Chen-Hui Mi, Xin-Ya Qi, Yan-Wen Zhou, Yan-Wen Ding, Dai-Xu Wei, Yong Wang

**Affiliations:** 1 Key Laboratory of Resource Biology and Biotechnology in Western China, Ministry of Education, School of Medicine, Department of Life Sciences and Medicine, Northwest University, Xi’an, 710069 China; 2 Department of Interventional Radiology and Vascular Surgery, Second Affiliated Hospital of Hainan Medical University, Haikou, China; 3 School of Clinical Medicine, Chengdu University, Chengdu, China; 4 Shaanxi Key Laboratory for Carbon-Neutral Technology, Xi’an, 710069 China

**Keywords:** Polyesters, Vascular tissue engineering, Drug delivery, Polyhydroxyalkanoates

## Abstract

Blood vessels are highly dynamic and complex structures with a variety of physiological functions, including the transport of oxygen, nutrients, and metabolic wastes. Their normal functioning involves the close and coordinated cooperation of a variety of cells. However, adverse internal and external environmental factors can lead to vascular damage and the induction of various vascular diseases, including atherosclerosis and thrombosis. This can have serious consequences for patients, and there is an urgent need for innovative techniques to repair damaged blood vessels. Polyesters have been extensively researched and used in the treatment of vascular disease and repair of blood vessels due to their excellent mechanical properties, adjustable biodegradation time, and excellent biocompatibility. Given the high complexity of vascular tissues, it is still challenging to optimize the utilization of polyesters for repairing damaged blood vessels. Nevertheless, they have considerable potential for vascular tissue engineering in a range of applications. This summary reviews the physicochemical properties of polyhydroxyalkanoate (PHA), polycaprolactone (PCL), poly-lactic acid (PLA), and poly(lactide-co-glycolide) (PLGA), focusing on their unique applications in vascular tissue engineering. Polyesters can be prepared not only as 3D scaffolds to repair damage as an alternative to vascular grafts, but also in various forms such as microspheres, fibrous membranes, and nanoparticles to deliver drugs or bioactive ingredients to damaged vessels. Finally, it is anticipated that further developments in polyesters will occur in the near future, with the potential to facilitate the wider application of these materials in vascular tissue engineering.

## Introduction

**Fig. 1:**
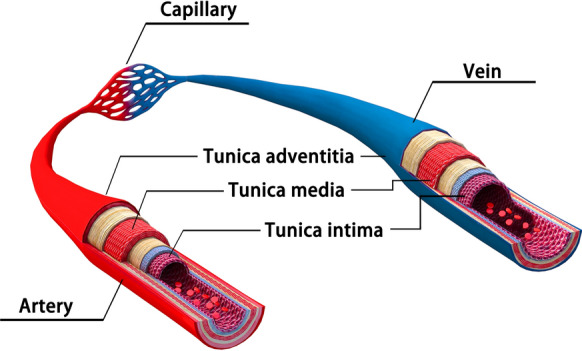
Vascular tissue and blood vessel wall structure

From a physiological perspective, mammalian vascular tissue is highly dynamic, providing both mechanical strength and a biochemical microenvironment conducive to the transport of blood and nutrients throughout the body [1]. Mammalian blood vessels can be classified into veins, arteries, and capillaries. The vessel walls are primarily composed of endothelial cells (ECs), vascular smooth muscle cells (VSMCs), and fibroblasts (FBs). While capillaries are mostly composed of only a monolayer of ECs, the vascular walls of arteries and veins have a three-layered structure [2]. The outer layer is composed mainly of FBs, which can provide the mechanical strength required by the vessels. The middle layer is formed by dense SMCs with fibrous or elastic bands of tissue. The inner lining is a continuous single layer of endothelial cells (ECs) directly connected to the basal membrane (Fig. 1) [3–5]. The spatial arrangement of these main vascular tissues varies, resulting in different thicknesses and compositions of the vessels [3, 6]. Vascular tissue fulfills a number of functions, including the transport of oxygen, nutrients, and metabolic waste products, as well as the conveyance of various pressure and shear forces. Moreover, it also regulates the composition of the blood and its osmotic pressure [7]. In addition, vascular tissue plays a pivotal role in immune regulation. According to their diameter, blood vessels can be divided into micro vessels (diameter < 1 mm), small vessels (1–6 mm), and large vessels (> 6 mm) [8].

Abnormalities in vascular tissue can give rise to vascular disease. Vascular disease represents one of the most significant contributors to impaired health and mortality in humans, encompassing a range of conditions including cardiovascular disease, cerebrovascular disease, vascular trauma, and vascular defects [9–11]. The mainstay of treatment for cardiovascular disease, which is the leading cause of mortality, is pharmacological and surgical intervention [12]. Nevertheless, it is evident that the current treatment modalities still have certain shortcomings, and transplantation is severely limited by the challenge of matching patients with suitable donors [13–15]. Consequently, vascular tissue engineering based on biological materials has attracted considerable interest from researchers. Implants obtained by combining microvascular systems with tissue engineering may anastomose with existing blood vessels in the host, thereby increasing vessel formation and improving viability [16, 17]. Initial research into vascular substitutes employed primarily Dacron (polyethylene terephthalate) and Goretex (polytetrafluoroethylene), which have been successfully implemented in the tissue engineering of large-diameter blood vessels. However, these materials face challenges of thrombosis and vascular occlusion when used for small-diameter vessels [18, 19]. For decades, small-diameter vascular grafts have been manufactured using polyesters.

**Fig. 2:**
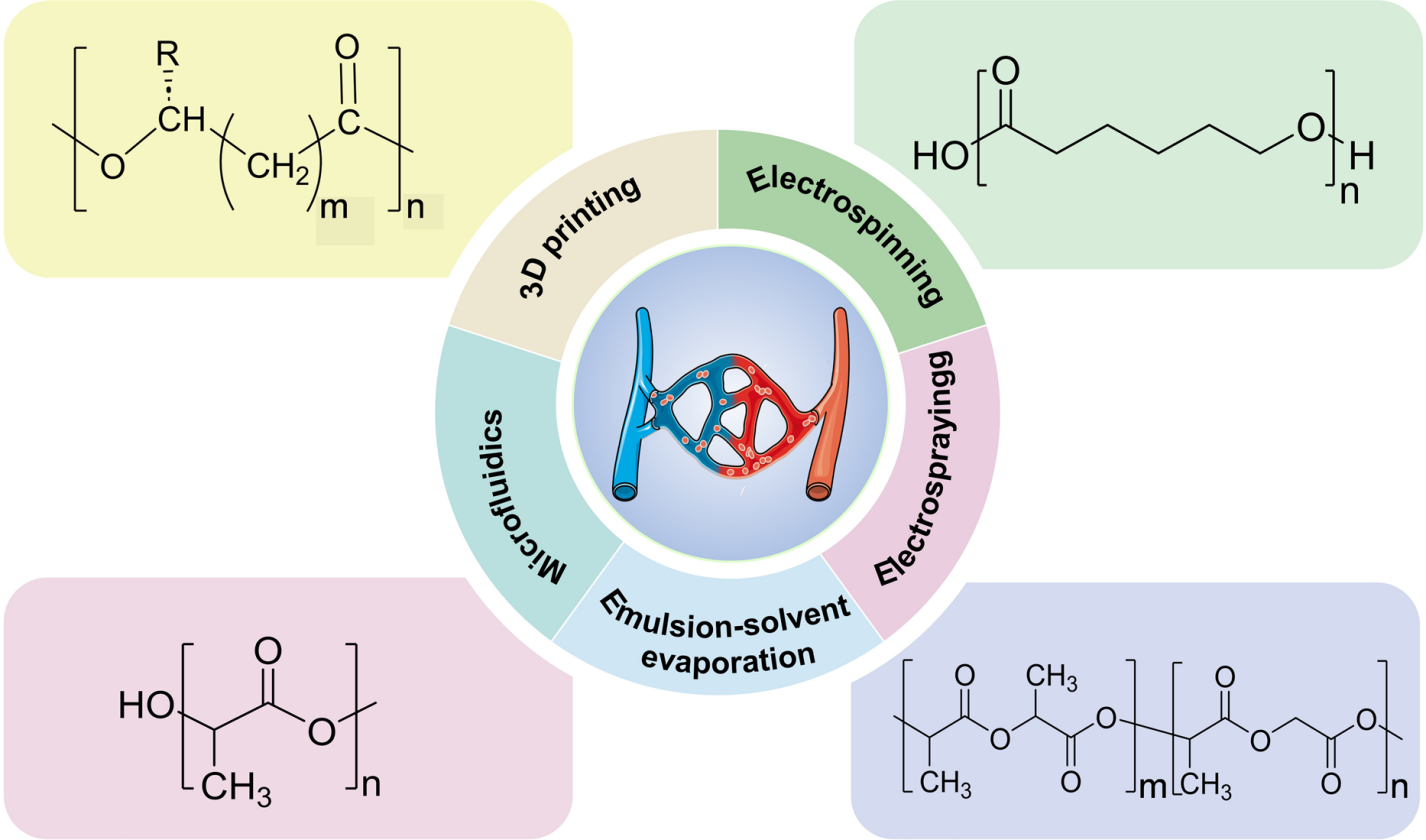
Schematic illustration of typical polyesters that are used in vascular tissue engineering

Synthetic biodegradable polymers, such as PHA, PCL, PLA, and PLGA, are widely used in vascular tissue engineering due to their excellent biocompatibility, safety, non-toxicity, reliable supply, and easy processing (Fig. 2) [20]. For example, PLA is degraded in the human body in 6–12 months, and its degradation product, lactic acid, is a natural mediator of carbohydrate metabolism, has favourable cellular interactions, and can be processed into tubes, thus representing a potential alternative to vascular grafts [21]. Polyesters can be degraded by chemical or enzymatic hydrolysis, and the resulting metabolites are completely non-toxic. Moreover, the degradation time and rate can be adapted by chemical modification, changing the ratio of monomers, or varying the length of the backbone [22–29]. Furthermore, in contrast to most conventional materials, polyesters are biodegradable, which plays a pivotal role in environmental protection. Pure polyesters can be combined with natural or other synthetic materials to enhance their biological activity and fulfill the requisite performance criteria for use in diverse tissues [30–32]. To date, polyesters have been extensively employed in the fields of bone, skin, cardiovascular, ophthalmic, and neural tissue engineering [24, 33–39]. It is of paramount importance that these materials possess robust mechanical properties when utilized in vascular tissue engineering, in order to maintain structural integrity in the face of various pressure and shear forces. Notably, polyesters can also be employed in vascular tissue engineering for the diagnosis and treatment of related diseases. For instance, they are used to promote endothelialization, alleviate the narrowing of the vascular lumen, regulate the rate of vascular repair, as well as forming both very small and large-diameter grafts. In short, polyesters are used in a wide range of vascular-related applications. Polyester-based scaffolds, when used as an alternative to vascular grafts, should have good biocompatibility, adequate mechanical strength and improved drug release kinetics. To meet these needs, the main strategies include: adjusting the monomer composition of the polyester-based material, chemically modifying the polyester-based material, incorporating bioactive factors or other materials, and continuously modifying and changing the processing method of the polyester-based material.

This review examines the distinctive properties and manufacturing technologies of polyesters for vascular tissue engineering applications. It outlines the specific applications of polyesters and their use in the treatment of vascular diseases, such as atherosclerosis and thrombosis, when employed as carriers for drug delivery systems (DDSs), over the past 5 years. Finally, we examine the limitations and shortcomings of polyesters for vascular tissue engineering. It is evident that these materials can be made better and safer through additional manufacturing process development and further clinical investigations.

## Classification and properties of polyesters

Polyesters, including PHA, PLA, PCL, and PLGA, play a crucial role in medical applications. These materials are characterized by their chemical groups, net charge, monomeric composition, and biodegradation behavior. Additionally, self-assembly properties, size, shape, and surface chemistry are important considerations in medical applications (Table 1) [40–44]. Among these properties, the polarity of the polymer is of fundamental importance to consider in medical applications such as vascular tissue engineering. It is evident that changes of hydrophobicity can have a profound impact on cell attachment, diffusion, and viability within targeted biological systems [45].

**Table 1:** Classification and properties of polyesters

Polyester material	Classification	Advantages	Disadvantages	Refs
PHA		Easy processing, good resistance to ultraviolet light, good biocompatibility	Inconsistent performance, complex processing, slow biodegradation	[46, 47]
	PHB	Brittle at room temperature, narrow processing temperature range, high crystallinity	Poor impact resistance, unstable melting state	[48–51]
	PHBV	High crystallinity, good biodegradability and biocompatibility	Low thermal stability, low toughness	[52–55]
	PHBHHx	High elongation at break, good flexibility, elasticity, and rigidity, biodegradability and biocompatibility	Loss of mechanical strength	[56–59]
	P34HB	Improved thermal stability and toughness	Low tensile strength, slow crystallization, low crystallinity, high possibility of post-crystallization	[60–62]
	PHBVHHx	High elongation at break, good flexibility, ductility, and rigidity, better biodegradability and biocompatibility	Low mechanical strength	[50, 58, 63, 64]
PCL	–	High toughness, low immunogenicity, good biocompatibility	High brittleness, low thermal stability, slow crystallization rate	[65]
PLA	–	High tensile and bending strength, simple processing	Poor toughness	[66, 67]
PLGA	–	Various processing forms, good water solubility, adjustable drug release	Hydrophobic, semi-permeable	[68, 69]

### PHA

Over the past few years, PHA has been extensively utilized in tissue engineering for the development of artificial blood vessels, heart valves, artificial cartilage, and wound dressings [70]. This is attributed to its advantages of facile processing, excellent UV resistance, insolubility in water, biodegradability, and biocompatibility [71–78]. Moreover, as a family of polyesters, PHAs have adjustable mechanical strength, which can be enhanced through chemical modification or blending with other polymers to obtain materials with improved mechanical performance [79–81]. In a study on the biocompatibility of PHA materials, FBs, ECs, and isolated hepatocytes exhibited high levels of adhesion when in direct contact with commonly used PHA materials such as poly(3-hydroxybutyrate) (PHB) and poly(-3-hydroxybutyrate-co-3-hydroxyvalerate) (PHBV) [82]. In addition to demonstrating biocompatibility for medical purposes, PHA materials must degrade within a clinically acceptable time frame to be used as temporary implant material [83, 84]. Notably, the PHA degradation products 3-hydroxybutyric acid (3HB) and 4-hydroxybutyrate (4HB) are metabolites that are naturally present in the human body. Among them, 3HB is a constituent of the blood, while 4HB is widely distributed in the brain, lungs, heart, liver, muscles, and kidneys [79, 85]. Qu et al. studied the in vivo tissue reaction and biodegradation of poly (3-hydroxybutyrate co-3-hydroxycaproate) (PHBHHx), PLA, PHB, PHBHHx, and polyethylene glycol (PEG) blends using the rabbit subcutaneous implantation assay. The findings indicated that PHB was degraded the most rapidly of all tested materials [58].

The mechanical properties of PHAs are determined by their monomer composition, chain length, as well as the distance between the side-chain and the ester bond. At room temperature, PHB is brittle, has poor impact resistance, and is highly susceptible to melting. Its processing temperature range is narrow (162–179 °C), and it exhibits crystallinity values of 60–80% [49, 51, 86]. PHBV is a highly crystalline polymer with a structure similar to PHB [50]. Kutrioka et al. observed that the crystallinity of PHBV was greater than 50% across the full range of 3-hydroxy valeric acid (3HV) content (0–95 mol%) [87]. By contrast, P34HB contains the more flexible 4HB monomer, which significantly enhances the material's toughness when copolymerized with the 3HB chain segment [60, 88]. However, as the 4HB content increases, the material's tensile strength decreases. The structural formula of a typical semi-crystalline polymer, P34HB, reveals that the spatial regularity of the P34HB chain is disrupted during the crystallization process due to the introduction of 4HB. The 4HB monomers are excluded from the PHB lattice, significantly reducing the crystallization capacity of PHB. This results in slower crystallization rates, lower crystallinity, and a higher likelihood of post-crystallization. Nevertheless, the incorporation of the 4HB monomer has been observed to enhance the thermal stability and toughness of P34HB [61, 89]. It is noteworthy that elongation at break for PHBHHx and PHBVHHx, respectively ranging from 108 to 270% and 277 to 740%, is incomparably larger than that of pure PHB, which falls within a range of 4.5–5.0% [50, 56]. These findings indicate that, in comparison to pure PHB, the introduction of 3HHx results in a reduction of mechanical strength, while simultaneously enhancing flexibility in multi-component copolymers. Furthermore, the incorporation of soft and amorphous monomers such as 3-hydroxy caproic acid (3HHx) and 3HV, has been demonstrated to enhance material toughness and rigidity [63, 90].

Furthermore, different types of PHAs exhibit varying degrees of biocompatibility and biodegradability. A study compared the biocompatibility of PHB and PHBV by examining their effects on cell-cycle progression. The results demonstrated that both materials significantly promoted cell cycle progression, with PHBV exhibiting enhanced efficacy in promoting cell adhesion and growth [91]. Furthermore, a separate study involving the injection of rabbit bone marrow cells bound to a PHBHHx triple stent revealed that its cell adhesion and proliferation ability was significantly superior to that of PHB, providing evidence for the superior biocompatibility of PHBHHx over PHB [59]. Qu et al. conducted an in vivo study to examine the tissue reaction and biodegradation of poly PHBHHx, PLA, PHB, PHBHHx (X), and PEG (E) blends using the rabbit subcutaneous implantation assay. The results demonstrated that the degradation rate followed the order of PHB < PHBHHx < PLA [92].

It is also important to consider the potential drawbacks of PHA. Despite its biodegradable nature, the market for this plastic remains limited due to its high production costs, inconsistent properties, and challenges in processing when compared to well-established conventional petroleum-derived plastics [47]. In addition, the in vivo explanation time of PHA is significantly longer than that of PLA and PLGA. Therefore, PHA is more appropriate for applications where biomaterials should be present in the body for an extended period and degrade slowly [46].

### PCL

PCL is a widely used biodegradable polymer that exhibits favorable mechanical properties and ease of processing, distinguishing it from other commonly utilized biodegradable scaffold materials such as PLA and polyglycolic acid (PGA) [93]. PCL has been employed in the fabrication of nanofiber scaffolds [94]. PCL is thermoplastic that exhibits semi-crystalline properties at 37 °C, imparting it with crucial attributes of flexibility and toughness [67]. Moreover, unmodified PCL degrades entirely in 2–3 years, enabling its use in cell-loaded patches capable of long-term integration into the myocardium with minimal scarring [66, 95, 96]. Furthermore, PCL is an excellent candidate for surface modifications to enhance hydrophilicity and improve biocompatibility, thereby facilitating enhanced cell adhesion [66]. Additionally, PCL can be blended with various polymers and other materials to modify its mechanical characteristics [67]. Finally, PCL exhibits low immunogenicity and favorable in vivo biocompatibility [95, 96]. In a study by Serrano et al., it was observed that L929 mouse FBs exhibited excellent adhesion, growth, viability, and mitochondrial activity during short-term culture on PCL membranes. Thus, PCL has emerged as a promising biocompatible scaffold material for vascular transplantation [97]. Nevertheless, PLA also has inherent limitations, including high brittleness, low thermal stability, and a slow crystallization rate, which restrict its applications [98].

### PLA

The currently available scaffolds constructed from soluble biomaterials have shown considerable potential in the field of vascular tissue engineering [99]. PLA has been extensively employed as a readily available bioabsorbable polyester [100–102]. A comparison of the properties of PLA with other widely used polymers (e.g., polyvinyl chloride (PVC), polypropylene (PP), and nylon) revealed that PLA has superior tensile and flexural strength [65]. Furthermore, PLA demonstrated significant potential for enhancement of its physical and mechanical properties and can be processed using simple conventional methods without requiring substantial energy or time inputs, making it a cost-effective and readily available polymer [103–106]. Furthermore, it is readily moldable and can be shaped into a variety of forms through injection molding. Ahuja et al. conducted a study to assess the effects of incorporating up to 5% of PCL and magnesium inclusions into PLA in a rat subcutaneous implantation model. The researchers characterized the cellular and tissue interactions, with a particular focus on the inflammatory response, angiogenesis, and envelopment. The results indicated that blood vessel formation in tissues containing PLA/PCL was significantly higher than in pure PLA only at 8 weeks. This suggests the need for more careful control of the material’s hydrophobicity for potential therapeutic angiogenesis. PLA is available in L-, D-, or L/D-form. It is semi-permeable to water and oxygen while exhibiting superior biodegradability compared to other biomedical polymers [107]. An increase in the porosity or surface-to-volume ratio of the polymer will result in an improved degradation rate [108]. However, the cost of manufacturing remains a critical issue that must be addressed to ensure commercial viability. Researchers must seek out cheaper substrates and improved microorganisms to enhance the production efficiency of PLA, with a focus of simultaneously decreasing cost and increasing quality. Furthermore, it is indispensable to develop new copolymers for blending with PLA to decrease costs and increase strength [65].

### PLGA

PLGA is a widely employed polymer in inhalable formulations due to its favorable mechanical and processing properties, presenting a significant opportunity for the pharmaceutical industry to develop innovative inhalable products [109]. PLGA can be machined into stents with a range of shapes and sizes, exhibits excellent water solubility, and enables controlled drug release [69]. The properties of PLGA copolymers are influenced by the ratio of PLA to PGA and their molecular weight, which affects their size, hydrophilicity, mechanical strength, and biodegradation rate [110–113]. Because PGA is hydrophilic and PLA is hydrophobic, PLGA copolymers with a higher PLA content demonstrate reduced hydrophilicity and lower water absorption, which results in a prolonged degradation period [114]. The degradation time of PLGA varies according to the proportion and molecular weight of the copolymer, with estimates ranging from several months to several years [27]. Additionally, PLGA is soluble in various organic solvents, including tetrahydrofuran, chloroform, ethyl acetate, and acetone [115, 116]. These distinctive features render PLGA highly versatile for numerous applications, particularly in controlled drug release [69]. Its biocompatibility and effective biodegradation have led to its wide use in tissue engineering. In a study by Bazgir et al., the biophysical properties and biocompatibility of three different biodegradable fiber scaffolds were compared, including PCL alone, PLGA alone, and coaxial scaffolds made of fiber cores and jackets from PCL and PLGA, respectively. The researchers discovered that PLGA membranes exhibited superior adhesion and enhanced cell proliferation. Furthermore, these biodegradable membranes exhibit good mechanical properties and can be used to construct vascular grafts [103]. Despite its numerous excellent properties, the hydrophobicity and semi-permeability of PLGA restrict its application in regenerative medicine, as PLGA does not absorb exudates and fails to provide a suitable humid environment [68].

## Preparation methods of medical polyester devices

Due to the thermoplastic properties of polyesters and their solubility in organic solvents, several processing methods have been explored to process these materials into systems suitable for a range of biomedical applications. The most common processing methods in current use include emulsion-solvent evaporation, electrospinning, electrospraying, and 3D printing (Table 2) [117–119]. In vascular tissue engineering, polyesters are frequently processed into diverse shapes and forms, including thin films, nanoparticles, fiber mats, and microspheres, among others [120–125]. When employed as a carrier for DDS, polyesters are often processed into nanoparticles and microspheres, which have a high porosity that allows them to carry drugs or other active factors while also facilitating cell adhesion and growth [126, 127]. When used as a graft for engineered vascular tissue, they are often processed into three-dimensional scaffolds. Moreover, they can also be processed into tri-layered scaffolds, which are closer to the natural structure of the vessel and thus alleviate the shortage of vascular grafts. Thus, polyesters can be manufactured into diverse forms using various processing techniques, thereby enhancing their suitability for vascular tissue engineering [121, 128, 129].

**Table 2:** Preparation methods and their characteristics

Method	Advantages	Disadvantages	Refs
Emulsion-solvent evaporation	Easily controlled process parameters, hydrophobic and hydrophilic drugs can be encapsulated	Compromises the integrity of biomolecules	[130]
Microfluidics	Small scale, low reagent consumption and waste, improved reaction efficiency, shortened analysis time, simple procedures, high portability	Long process of molecular diffusion	[131]
Electrospinning	Large surface area and micro-porosity, controllable morphology	Poor cell penetration, low carrying capacity, low mechanical strength	[132, 133]
Electrospraying	High loading efficiency and narrow particle size distribution, no nee for surfactants	Destruction of biomolecules under certain conditions	[134, 135]
3D printing	Rapid prototyping, faster small batch production, flexibility and space for innovation, easy process, broad affordability	High cost, low preparation speed	[136, 137]

### Emulsion-solvent evaporation

The emulsification-solvent evaporation method is widely used to obtain nanoparticles and thin films. When used in vascular tissue engineering, the nanoparticles can encapsulate hydrophobic and hydrophilic drugs, while the thin films can reduce the risk of thrombosis by influencing cell adhesion and proliferation [130]. Typically, the drug is emulsified or dissolved in a hydrophobic solvent and then emulsified in a continuous aqueous solution. Subsequently, evaporation of the solvent is used to solidify the material into particles, which are then washed with distilled water, collected by centrifugation, and freeze-dried, resulting in a formulation suitable for long-term storage [138, 139]. For example, a standard two-emulsion scheme for preparing PHB nanoparticles first involves ultrasonication and stirring, whereby an aqueous phase containing drugs and the emulsifier is added to the organic polymer solution. The resulting water-in-oil (w/o) emulsion is poured into a second aqueous phase containing a hydrophilic polymer (such as polyvinyl alcohol) (PVA) to form a w/o/w emulsion. This system is stirred until the organic solvent completely evaporates, followed by centrifugation and re-suspension in the aqueous phase. Recently, folate-coupled PHB nanoparticles were prepared using this method and loaded with anticancer drugs [140]. It is necessary to apply shear forces when the organic phase contains internal aqueous droplets comprising biomolecules. However, these forces have the potential to compromise the structural integrity of biomolecules, resulting in the loss of biological activity. Furthermore, organic solvents used for homogenization may have a detrimental effect on the structure of biomolecules [130].

### Microfluidics

Microfluidics is a scientific and technological discipline focused on systems that manipulate small volumes of fluids or suspensions within channels from tens to hundreds of microns in diameter [141]. Extensive research and constant development of microfluidic platform technologies have led to the continuous exploration and application in vascular tissue engineering. Microfluidics are often used to process polyesters into microspheres, mainly for the delivery of drugs and other bioactive factors. The prepared composite microspheres can reduce the initial release of encapsulated substances and achieve slow release to better treat diseases [142, 143]. Consequently, this processing method holds promise for application in vascular tissue engineering. The inherent small scale of microfluidics provides competitive advantages over traditional methods, including reduced reagent consumption and waste, enhanced reaction efficiency, shortened analysis time, simplified procedures, and increased portability [131]. Furthermore, various hydrodynamic, electrodynamic, acoustic, magnetic, centrifugal, and capillary forces can be harnessed to manipulate particles or biological entities, enabling phenomena such as separation, enrichment, focusing, alignment, visualization, capture, classification, and isolation [144–146]. However, because of the micrometer scale and laminar flow of microchannels, mixing can only be accomplished through a long-term molecular diffusion process [131]. Although the microspheres produced by microfluidic technology have a high drug encapsulation rate, reduced drug loss, and can easily be made sterile, further research is required to achieve a consistent droplet size [147–149].

### Electrospinning

Electrospinning enables the fabrication of polyesters with a structure similar to the walls of a natural vessel, including double- or even triple-layer vascular graft substitutes and fiber mats with specific structural arrangements. This alleviates the shortage of grafts in clinical practice, while also allowing the targeted application of drugs (such as heparin) to the damaged site, effectively relieving and treating vascular diseases [129, 150, 151]. In its simplest form, electrospinning involves a high-voltage DC power supply, injection pump, spinneret (typically a blunt hypodermic needle), and a collector [152–154]. In vascular tissue engineering, a rotating mandrel is often utilized as the collector to manufacture tubular grafts [152]. This technique offers flexible production and scaling capabilities, large pore size and micro-porosity, large surface area, ease of tuning the morphology, and intriguing mechanical properties typically unattainable through standard techniques [155, 156]. These attributes have established electrospinning as an essential technology in the field of polyester fabrication. Notably, fiber pads can effectively mimic the natural structure of biological tissues [156, 157]. In addition, the increase in the surface area of nanofiber pads can imbue the material with new physical and chemical properties [158]. However, despite its wide range of applications and apparent advantages, electro-spun scaffolds have two significant practical limitations—poor cell penetration and low load-carrying capacity. Additionally, the mechanical strength of electro-spun scaffolds is low [132, 133, 159].

### Electrospraying

In electrospraying, micro- and nanoparticles (spheres or capsules) can be obtained from polymers dissolved in a conductive solvent. The technique is based on principles similar to electrospinning [160]. At the same time, there are differences in the solution (concentration, solvent, and viscosity) and manufacturing process parameters, including flow rate, distance from tip to collector, and voltage [161]. Additionally, during the formation of a jet from an electrospray Taylor cone, parameter changes will cause the jet to break into droplets, resulting in particles of varying sizes and shapes [134, 160, 162]. Compared with other technologies, micro- and nanoparticles obtained through electrospraying exhibit higher loading efficiency and a narrower particle size distribution [134]. Furthermore, the need to separate particles from dispersed solutions and even use non-degradable surfactants in some manufacturing techniques can pose additional challenges. Electro-spraying allows particle fabrication in just one step without requiring surfactants [134, 163]. While many studies have reported minimal damage to biomolecules during electrospraying, significant loss of biological activity can occur under certain conditions [135].

### 3D printing

As an additive manufacturing technology, 3D printing offers advantages such as rapid prototyping, faster small batch production, flexibility and space for innovation, reduced complexity, and widespread affordability [136]. Thus, 3D printing has the potential to replace traditional processing methods for widespread tissue engineering use. Broadly speaking, the main 3D printing technologies are (i) selective layer/laser sintering (SLS); (ii) fused fabrication (FFF), also known as fused deposition modeling (FDM) or fused polymer deposition; (iii) stereolithography; (iv) digital light processing; (v) multi-jet/inkjet 3D printing; and (vi) electron beam melting [164, 165]. Currently, the main technologies used for biodegradable polymers are SLS and FDM. However, the high cost and low speed of 3D printing technology still limits its use. Whereas the average cost of a 3D printer has declined recently, material and maintenance costs remain high. In addition, 3D printers are still slow compared to traditional manufacturing methods. Hence, more experimentation and innovation are needed to reduce the price and increase the development speed of 3D printing technology [137]. In vascular tissue engineering, 3D printing can be used to fabricate a bionic scaffold of blood vessels, which can effectively promote vascularization by fixing vascular endothelial factors on the surface of the 3D-printed structure [166]. In addition, because the contact area between the stent and the damaged site is small, the inflammatory response can also be reduced to a certain extent [167].

## Applications of polyesters in vascular tissue engineering

Polyesters can be effectively adapted through various processing techniques to create scaffolds with suitable hemocompatibility, biodegradability, and variable mechanical properties suitable for vascular tissue engineering. These scaffolds can be utilized not only to replace large and small vascular grafts, but also as drug delivery carriers to facilitate the targeted transport of exogenous active ingredients, thereby promoting vascular repair to treat vascular diseases [168]. This review details the recent advances in the application of polyesters in vascular tissue engineering from 2019 to 2024 (Table 3).

**Table 3:** Applications of polyesters in the vascular system

Polyester material	Other matrix materials	Active substance	Medical devices	Functions	Applications	Ref
P3HB10U (PHA)	PEGDA	Graphdiyne	Tubular scaffold	Cell infiltration, tissue regeneration, higher patency rate	Small-diameter vascular graft	[169]
PHBV (PHA)	PCL	VEGF, bFGF, SDF-1α	Tubular scaffold	Higher primary patency	Small-diameter vascular graft	[170]
PHBV (PHA)	–	HDMECs, HDFs	Scaffold	Promote angiogenesis	Tissue engineering	[171]
P3HB (PHA)	–	–	Film	Hemocompatibility	Vascular implants	[120]
PHBV (PHA)	PCL	VEGF, bFGF, SDF-1α, Heparin, Iloprost	Scaffold	Fully endothelialized, highly vascularized	Reconstruction of small arteries	[172]
P4HB (PHA)	PLLA	Sirolimus	Scaffold	Reduce inflammation, promotes tissue growth	Interventional application	[173]
PCL	–	Iloprost	Prostheses	Better regenerative potential	Vascular graft	[174]
	PU	–	Scaffold	Mechanical support and stability	Vascular graft	[129]
	COL, GEL	–	Scaffold	good biocompatibility and sufficient mechanical strength	Vascular graft	[151]
	–	H_2_S donors	Scaffold	Promote angiogenesis	Tissue engineering	[175]
	GEL	Carbon nanotubes	Scaffold	Biomimetic mechanical properties support cell proliferation	Vascular tissue engineering	[176]
	–	ODECs, ODMCs	3D-scaffold	New cell source	Human macrovascular models	[177]
	–	Fucoidan	Fibrous meshes	Promote vascularization	Tissue engineering	[178]
	–	pCMV-VEGF165-plasmid	Microfiber scaffold	Improve vascularization	Vascular tissue engineering	[179]
	PEO	Wintergreen oil	Scaffold	Antioxidant and blood compatibility	Synthetic small-size vascular grafts	[180]
	GelMA	–	Scaffold	ECs axially and SMCs circumferentially	Tissue-engineered vascular grafts	[181]
	–	–	Membranes	Support angiogenesis	Tissue engineering	[182]
	SF	Human amniotic membrane	Scaffold	Endothelialization, resisting collagen deposition,	small-diameter vascular grafts	[183]
	PC-BU	SDF-1α	Scaffold	Self-healing capacity	Living arteriovenous fistula	[184]
	–	–	Microfiber scaffolds	High cell viability and coverage	Small-diameter vascular graft	[185]
	GT	PDA	Fiber mats	Microvascular development	Functional tissue regeneration	[186]
	–	SF-Hep	Scaffold	Antithrombosis and long-term patency	Small-diameter vascular graft	[187]
	–	S-nitrosated keratin	Scaffold	Rapid endothelialization and vascular remolding	Small-diameter vascular graft	[188]
PCL	–	S-NO-HSA	Patches	Rapid surface endothelialization and late migration of SMCs	Small-diameter vascular graft	[189]
	–	Heparin	Core–shell fibrous	SMCs regeneration, endothelialization, high patency rate	Vascular tissue regeneration	[150]
	PU	Heparin, aspirin	Scaffold	prevent acute thrombosis, promote intimal construction	Small-diameter vascular graft	[190]
	–	Heparin, ECM, VEGF	Scaffold	cells infiltration	Small-diameter vascular graft	[191]
	–	Catechol	Scaffold	Anti-inflammation; antithrombogenicity	Vascular graft	[192]
	–	Keratin	Scaffold	Rapid complete endothelialization, reduce SMC proliferation	Small-diameter blood vessel substitutes	[193]
	–	Keratin, Cu (II)	Fiber mats	Vascular remodeling	Vascular tissue regeneration	[122]
	Chitosan	Cu (II)	Fiber mats	Enhance angiogenesis	Tissue engineering	[194]
PLA	PGS	oxygen plasma	Scaffold	Improvement in blood compatibility and cell growth	Vascular tissue regeneration	[195]
	–	Fe@C NPs	Scaffold	Destruction of atherosclerotic plaque structures	Anti-atherosclerotic agents	[196]
	PHD	Heparin	Scaffold	Promote a stable and functional endothelial cell layer	Small-diameter vascular graft	[197]
	HA	ECM, hCOLIII	Scaffold	Enhanced endothelialization, suppressed inflammatory response, and superior thromboprotection	Cardiovascular stents	[198]
PLGA	GEL	PEGylated curcumin	Core–shell nanofibers	Enhanced vascularization	Controlled nanotherapeutic release	[199]
PLGA	PLA, GEL	–	Tubular scaffold	Promote SMCs and ECs proliferation	Vascular tissue engineering	[200]
	–	Heparin and SDF-1α	Scaffold	Anticoagulation, endothelialization	Small-diameter vascular graft	[201]
	CS	–	Nanofibrous membrane	Shape-memory	Small-diameter vascular graft	[202]
	–	–	Hierarchical vascular graft	Mechanical properties, cytocompatibility, and endothelialization	Vascular replacement	[203]
	EGFP-EGF1	Dir	Nanoparticle	Target delivery	Targeted therapy for atherosclerosis	[121]
	PEG	Rapamycin	Nanoparticle	Safer and more efficient	Therapy of atherosclerosis	[204]
	–	Desferrioxamine	Nanocomposite gels	Promote greater angiogenesis	Scaffolding biomaterials for vascularization	[205]
	–	Rapamycin- or necrostatin-1	Nanoparticle	Inhibit inflammatory reaction and foreign body reaction	Potential clinical application	[206]
	–	Rapamycin	Scaffold	Decrease neointimal thickness	Vascular patches	[207]
	–	Superparamagnetic iron-oxide nanoparticles	Scaffold	Evaluate TEVG remodeling	Ensure proper prosthesis engraftment	[208]
	–	BOD-L-β-Gal	Nanoprobe	Real-time monitoring of β-Gal	Early diagnosis and therapy of atherosclerosis	[209]
	–	Magnesium hydroxide	Drug-eluting stents	Repressing endothelial activation	Safety issues in polymer-based implants	[210]
	PEG	CRISPR-Cas9 plasmid DNA	Nanoparticles	Genome editing	Gene delivery, gene therapy	[211]
	–	Rapamycin	Drug-eluting stents	Endothelialization potential, resistance to restenosis	Drug release	[212]
	–	VEGF, ZnO	nanofibers	Angiogenesis, antibacterial, bioactive membranes	Against infectious wounds	[213]
PLGA	HA, SA, CaCO3	Rapamycin	Hydrogel	Inhibiting neointimal hyperplasia after vascular interventions	Drug delivery	[214]
	–	IGF-1	Microspheres	Angiogenesis	Flaps/treatment of traumatic injury	[123]
	–	Bevacizumab	Nanoparticles	Targeted delivery to atherosclerotic lesions	Targeted drug delivery	[215]
	–	VEGF, HSA	Microspheres	Angiogenesis	Treatment of ischemic tissue or wounds	[128]
	–	NL-1	Nanoparticles	Prevention of peroxide generation, reduction of apoptosis	Treatment of cerebral ischemia/reperfusion injury	[216]
	–	Verteporfin, CB-839	Microparticles	Combined delivery of drugs	Pulmonary hypertension	[217]
	–	H_2_S-releasing aspirin derivative	microspheres	Inhalable and efficacious H_2_S donor	Treatment of PAH	[218]
	–	HVE-cad-Fc	Porous scaffold	Regulating the secretion of hMSCs	Vascularization in tissue engineering	[219]
	–	MicroRNA-145	Nanoparticles	Attenuating intimal hyperplasia	DDSs, vein graft	[220]
	–	Sialyl-Lewis-acid	Nanoparticles	Enhancing the margination of nanoparticles	Targeted drug delivery to vascular tissue	[221]
	–	Quercetin	Nanoparticles	Reducing restenosis post-angioplasty	Peripheral artery disease	[222]
	–	1α,25-dihydroxy vitamin D3	Nanoparticles	Increasing vessel area, decreasing neointima area/media area ratio and cell density	AVF stenosis treated with PTA	[223]
	–	Tacrolimus mycophenolate mofetil, prednisolone	Microspheres	Immunosuppression	Vascularized composite allotransplantation	[224]
	–	Paclitaxel	Nanoparticles	Tissue penetration	In-stent restenosis	[225]

### PHA-based materials for vascular applications

PHA are natural polyesters produced by a wide array of microorganisms as an energy and carbon storage compound within the cell [226, 227]. As a consequence, PHA has intrinsic biocompatibility, biodegradability, and safety. From a chemical perspective, PHA is an aliphatic polyester that can be classified into three categories based on the number of side-chain carbon atoms [228–231]. One outstanding feature of PHA used as a polymer scaffold is that it can displace petrochemical plastics, thus achieving environmental protection [22]. In addition to its intrinsic biodegradability, PHA also has variable mechanical properties, which can be adjusted by varying the monomer composition, chain length, as well as the separation between the ester bond and the side-chain [23, 24]. The polymer scaffold formed by PHA also has an appropriate porosity, allowing the passage of gas and water to promote the growth, proliferation, and differentiation of cells, which is beneficial to the repair of vascular tissue defects [232, 233].

To overcome the hydrophobic nature of PHA when applied to vascular tissue, Hou et al. combined poly(3-hydroxybutyrate-co-3-hydroxy-10-undecenoate) (P3HB10U) with polyethylene glycol diacrylate (PEGDA) and added graphdiyne (GDY) nanoparticles to the gel, which enhanced the mechanical strength [169].

**Fig. 3:**
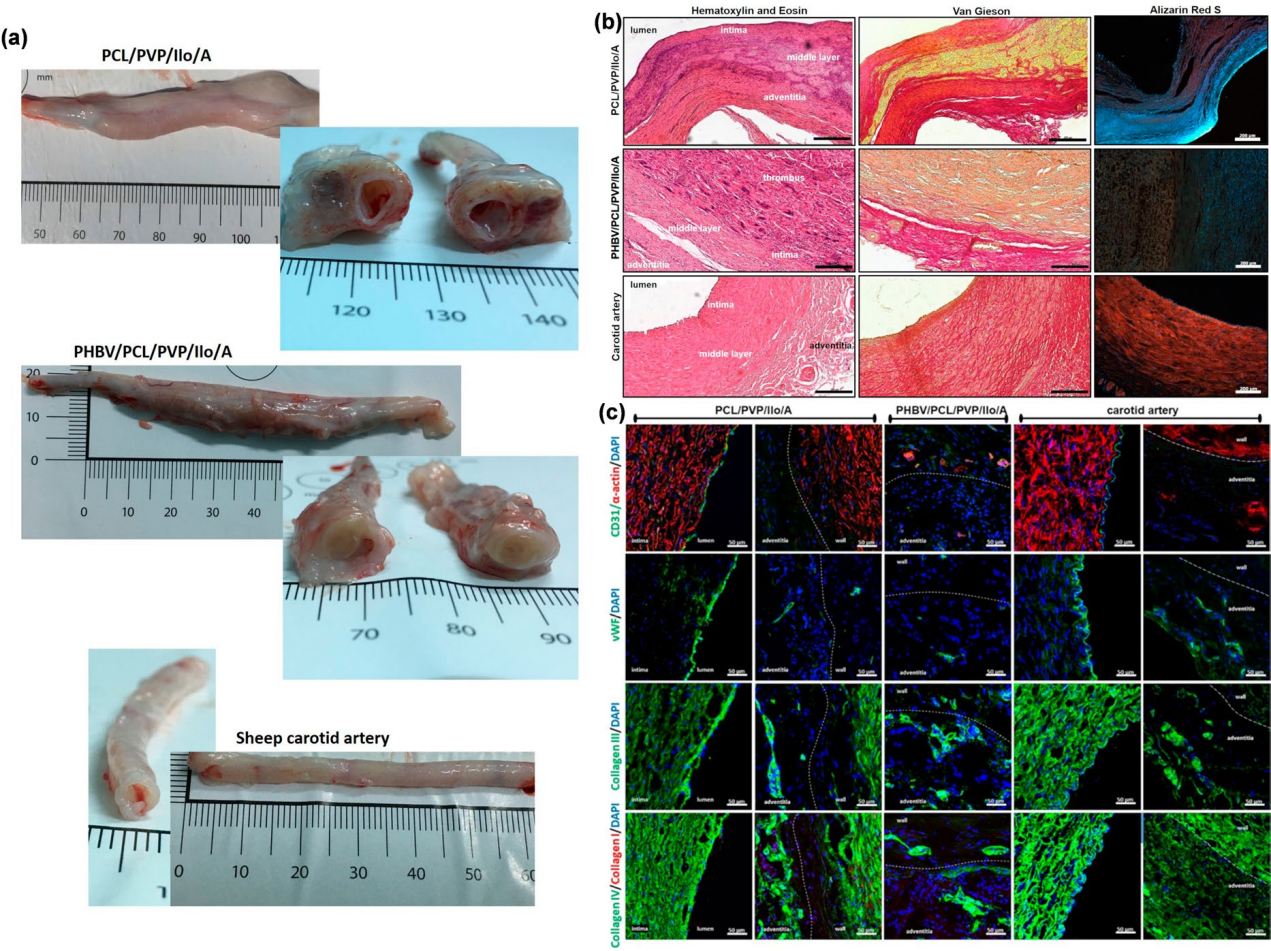
**a** Macroscopic examination of explanted vascular PCL/PVP/Ilo/A and PHBV/PCL/PVP/Ilo/A prostheses after 6 months of implantation in the carotid artery in sheep and the contralateral carotid artery. **b** Morphological observation of the explanted PCL/PVP/Ilo/A and PHBV/PCL/PVP/Ilo/A prostheses after 6 months of implantation into the ovine carotid artery. Scale bar = 200 µm **c** Evaluation of the cell density and collagen deposition of explanted PCL/PVP/Ilo/A and PHBV/PCL/PVP/Ilo/A prostheses 6 months after implantation. Representative immunofluorescence-stained cross-sections of the explanted prostheses. Dashed lines are used to distinguish the boundaries between the walls of the prosthesis (wall) and surrounding tissue (adventitia). Scale bar = 50 µm. (reprinted with permission from Ref [174])

The study found that in the early stages, the surface of the PHA/PEGDA-GDY organic hydrogel vascular graft was smoother, with reduced aggregation of blood platelets and blood coagulation. This reduced the risk of thrombus formation, offering a more suitable endothelial microenvironment than the control [169]. In cell-culture experiments, PHA/PEGDA-GDY effectively promoted the proliferation of ECs and SMCs. In a rabbit left carotid artery model, the vascular wall thickness of the PHA/PEGDA-GDY scaffold was closer to that of the natural vessel, providing a better immune microenvironment for endothelial cell aggregation and tissue regeneration. However, it is worth noting that the mechanical strength of this scaffold is slightly lower than that of the regular vessel, and further efforts are needed to close this gap [169]. Antonova et al. validated the feasibility of using PHA materials as vascular grafts in the carotid artery of sheep by preparing PHBV/PCL two-layer scaffolds via emulsion electrospinning, which contained vascular endothelial growth factor (VEGF) in the inner layer, as well as stromal cell-derived factor 1α (SDF-1α) and essential fibroblast growth factor (bFGF) in the outer layer [170]. When heparin and iloprost were conjugated onto the stent, the anti-thrombotic performance was improved compared to a poly(tetrafluoroethylene) (ePTFE) vascular stent [172]. The 24-h patency of PHBV/PCL was 62.5% (5/8), which decreased to only 50% (4/8) after 18 months, while all the control groups were blocked within 24 h [172]. In addition, the regenerated artery showed a three-layer structure similar to natural vessels, complete endothelialization, and high vascularization, whereby it was filled with vascular smooth muscle cells and macrophages (Fig. 3) [172]. After said study, the Antonova team contrasted the mechanical capacities, blood compatibility, long-term patency, and tissue regeneration characteristics of the DDS made of iloprost-loaded PCL stents and PHBV/PCL stents in a sheep implantation carotid artery model, which indicated that the regenerative potential of PCL alone was better than that of PHBV/PCL and was more suitable for clinical use [174]. PHBV/PCL materials prepared by electrospinning also showed significant vasoactivity in the chorioallantoic membrane of fertilized chicken eggs [171]. In addition to animal experiments that demonstrated the in vivo applicability of PHA materials in vascular tissues, PHA materials were also reported to promote the proliferation of vascular tissue-related cells as well as the formation of a monolayer of endothelial cells in cell culture experiments [120, 234]. Other types of PHA materials, such as PHBHHx and P34HB, can effectively promote and induce angiogenesis at the sites of bone damage and thus hold dual application potential in vascular and bone tissue engineering [235, 236].

### PCL-based materials for vascular applications

A prominent feature of PCL is its extreme mechanical toughness and excellent elasticity, combined with, biocompatibility and biodegradability [25, 26]. Nevertheless, its biodegradation rate is much lower than that of other organic polymers, with degradation requiring 2–4 years [237]. Due to its slow degradation, scaffolds made of PCL have great potential in promoting vascular tissue regeneration [238]. However, the low bioactivity and hydrophobicity of PCL lead to low cell activity and are not conducive to cell adhesion/proliferation [239]. To remedy this shortcoming, PCL can be combined with other polymers to maintain wound moisture, improve the antibacterial performance, and promote tissue regeneration [31, 32, 240, 241].

**Fig. 4:**
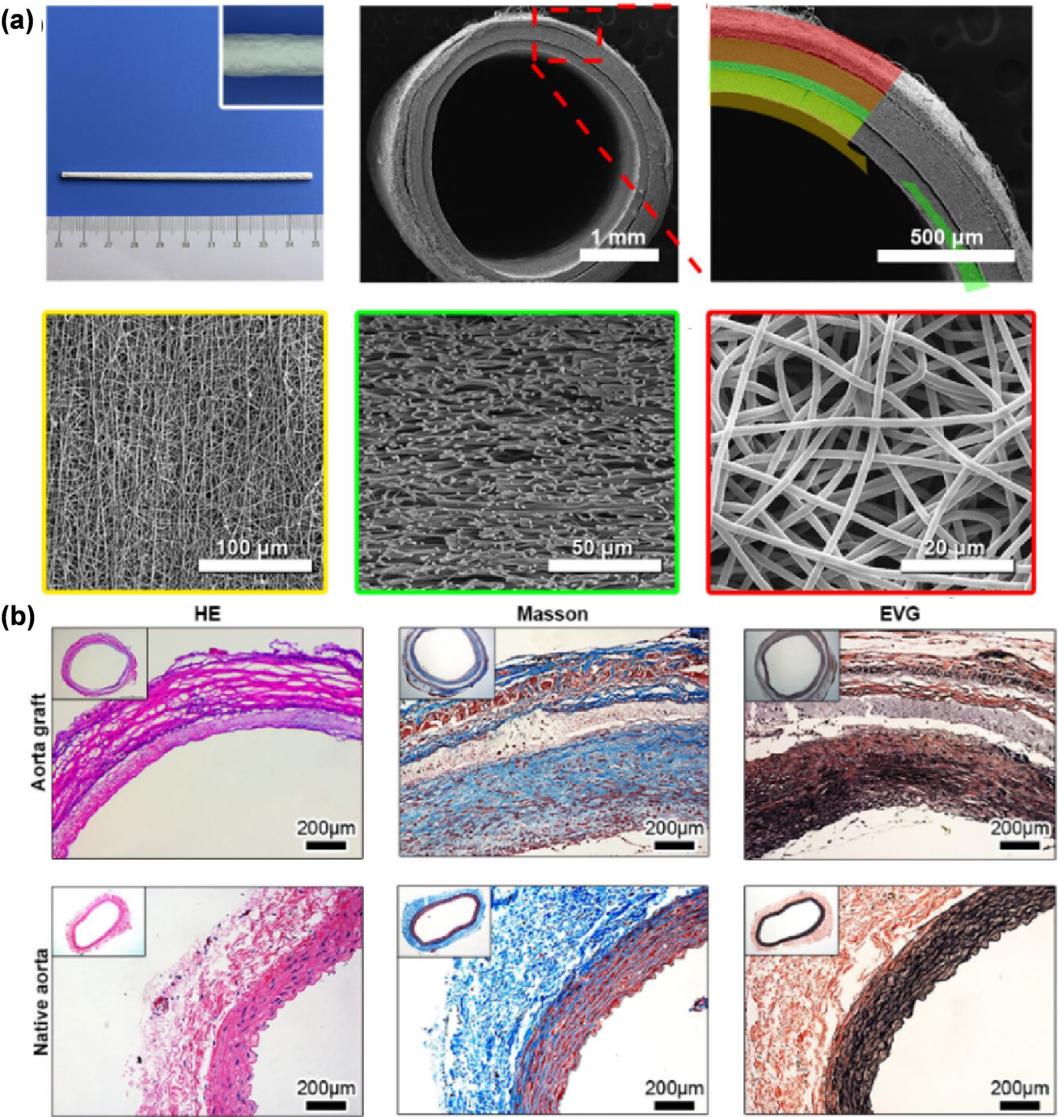
**a** Construction of the biomimetic blood vessel **b** Cross-section 180 days after implantation (reprinted with permission from Ref. [129])

When PCL -based materials are used in vascular tissue engineering, they can offer efficient mechanical support and stability as well as reduce platelet adhesion and the risk of thrombosis. Bao et al. designed a three-layer scaffold structure to imitate the native three-layer structure of arteries. The inner layer consisted of PCL, and the fiber direction was consistent with the blood flow, effectively preventing platelet formation. The middle layer was composed of PCL and polyurethane (PU), which provided mechanical support by SMCs and an anti-swelling PU network. Irregularly arranged PCL fibers formed the outer layer that accelerated the growth of nerves and pericytes, resulting in a biomimetic vascular autograft that can achieve the physiological adjustment of the aorta (Fig. 4) [129]. Hu et al. also designed three-layered scaffolds containing different materials, all of which showed excellent mechanical properties and the potential to promote tissue growth [151, 242]. The rate of cell proliferation is one of the critical factors in the repair of vascular tissues. Studies have confirmed that PCL scaffolds can support cell proliferation, thereby promoting endothelialization and vascularization at the injured site, which was verified by both in vitro and in vivo experiments [175–179]. In addition to being used purely in vascular tissues, PCL polyester-based scaffolds can be used to promote bone repair by promoting vascular proliferation and vascularization at the injured site [166, 243–251]. When PCL scaffolds are combined with polymer materials, the physical and chemical characteristics of the scaffolds can be effectively improved. More ideal effects can be achieved when the scaffolds are combined with other materials. When wintergreen oil (methyl salicylate) is incorporated into the electrospinning process, it can effectively prevent thrombus formation [180]. Most PCL stents can be used to create small-diameter vascular grafts, but Meijer et al. developed a polycaprolactone-bis urea (PCL-BU) scaffold that can be used as a human large vessel model of 3D scaffolds [177]. PCL has gradually become a favored vascular tissue engineering scaffold in recent years due to its unique properties.

### PLA-based materials for vascular applications

PLA is the polyester generated by the polymerization of lactide and lactate monomer, with outstanding characteristics of amphiphilicity and unique mechanical properties [100, 252]. Among them, a higher proportion of PLLA can effectively enhance the mechanical flexibility of the polymer scaffold [253]. It is important to note that the degradation time of PLA can be adjusted according to the molecular weight. PLA with a lower molecular weight degrades more rapidly, and the carbon dioxide and water produced by the degradation of PLA have no harmful effects on living organisms, in contrast to other polyester-based materials [253, 254]. A variety of PLA scaffolds, including tubular, cylindrical, and porous varieties, can be produced through different processing techniques and may serve as a potential substitute for vascular grafts and drug delivery systems for vascular tissues [255, 256].

**Fig. 5:**
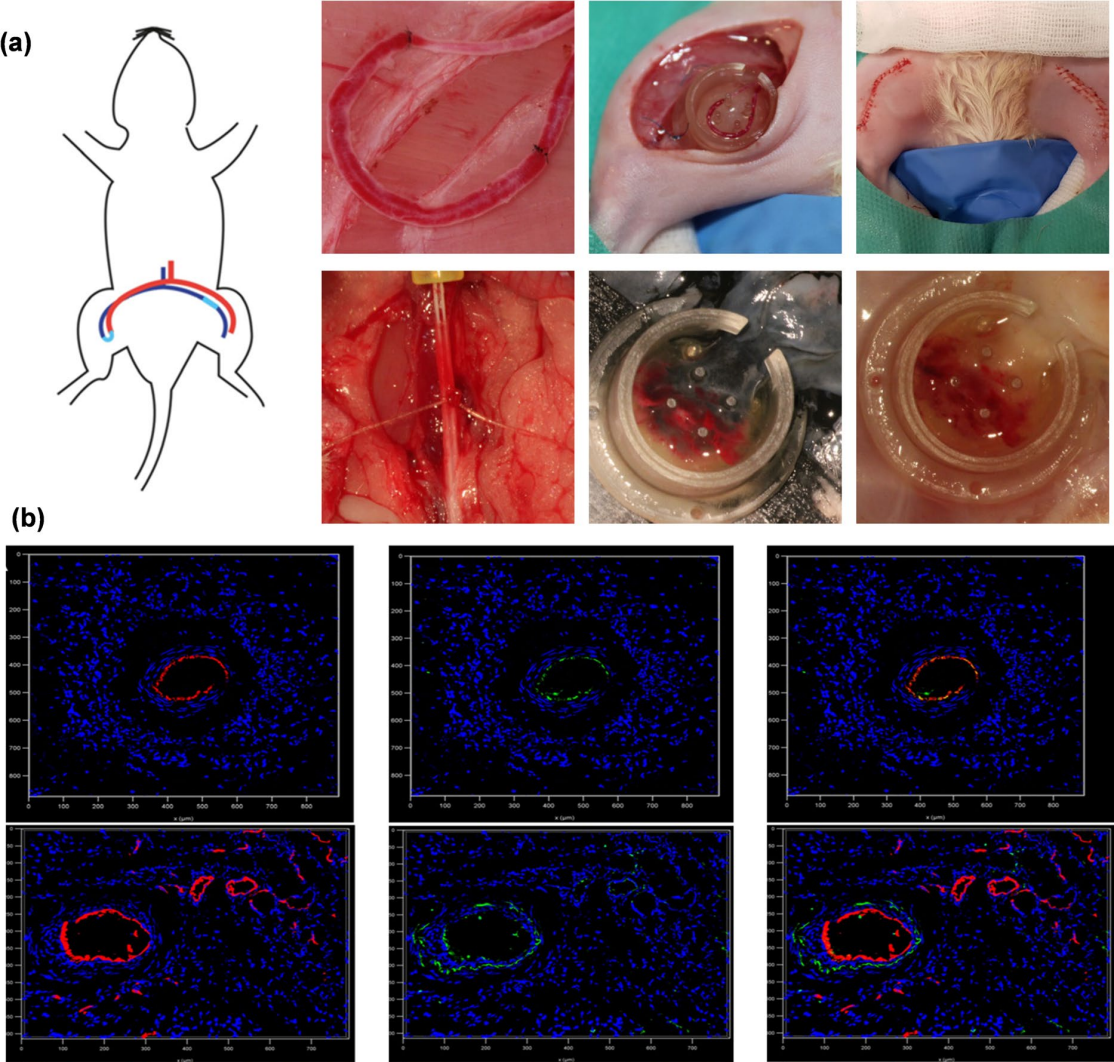
**a** Experimental setup and AV loop procedure. **b** Immunostaining for CD31 (shown in green) revealed colocalization with MHI148-PEI (shown in red). The main arterial inflow tract in PLA-based tissue was constructed after seven days. After 28 days, vascularization of the PLA-based construct was observed by CD31 staining and MHI148-PEI perfusion. (reprinted with permission from Ref. [259])

To demonstrate the angiogenic ability of PLA scaffolds, Koepple et al. compared the shear stress-induced axial vascularization ability of a homogeneous PLA microporous matrix and proven collagen-elastoprotein (CE) matrix [257–259]. Compared with PLA scaffolds, the total cell count in CE scaffolds was higher, and cell infiltration increased with time only in CE scaffolds [259]. Nevertheless, the total cell count in the PLA scaffold remained stable over time, and the maximum vascular density of the PLA scaffold reached a higher peak and remained stable toward the periphery [259]. This study not only demonstrated the applicability of the PLA scaffold in inducing angiogenesis from the atrioventricular ring, achieving soft tissue maturation and maintaining a microenvironment appropriate for axial vascularized tissue engineering, but also enhanced our understanding of angiogenesis in artificially engineered soft tissue structures by using PLA scaffolds with low spontaneous fluorescence (Fig. 5) [259]. Studies have shown that PLA yields better scaffolds for vascular tissue engineering when combined with other materials. When PLA is combined with acrylonitrile butadiene styrene (ABS) or poly glycerol sebacate (PGS), it can effectively improve the mechanical strength of vascular grafts to more closely resemble the performance of natural blood vessels. When combined with acellular dermal matrix (ADM), it was found to significantly improve the expression levels of VEGF and transforming growth factor-β (TGF-β). When combined with carbon-coated iron nanoparticles (Fe@C NPs) and cobalt-chromium (CoCr), it can activate macrophages and achieve an anti-atherosclerotic effect. When combined with bioglass (BG), the resulting polymer scaffolds have a positive impact on the viability of endothelial cells. When PLA and calcium phosphate nanoparticles were combined, the migration of rat endothelial progenitor cells was significantly increased, resulting in an increase in pro-vascular substances and upregulation of pro-inflammatory proteins [167, 195, 196, 260, 261]. In addition, PLA scaffolds can be used for the repair of vascular tissues by promoting early vascular generation, treating vascular stenosis, and creating branched hollow scaffolds for angiogenesis [262–264]. As a drug with anticoagulant effects in vivo and in vitro, heparin has been widely used in various cardiovascular diseases in the clinic. Similarly, it can also be used as one of the primary drugs in DDS for vascular tissue engineering [150, 190–192, 197]. Caracciolo et al. developed a PLLA and segmented polyurethane (PHD) blend-based electrospun double-layered small-diameter vascular graft and evaluated its ability to support the growth of human umbilical vein endothelial cells (HUVECs) [197]. Heparin was selected as an anticoagulant because it can accelerate the growth of HUVECs and suppress the excessive proliferation of smooth muscle cells, thereby avoiding neovascularization and restenosis [197].

### PLGA-based materials for vascular applications

PLGA is synthesized via the irregular polymerization of lactic acid and glycolic acid. Because the properties of the two monomers are different, the mechanical, physical, and chemical properties as well as degradation time of PLGA can be varied by changing the monomer ratio [27–29]. It is noteworthy that PLGA is not only biodegradable in the environment, but can also be degraded into esters in mammalian tissue and thus be excreted out of the body [265]. Despite the many good properties of PLGA, its hydrophobicity and semi-permeability have limited its applications in regenerative medicine. Hence, PLGA cannot absorb exudates and also cannot provide a suitable wet environment [68]. Therefore, PLGA is often combined with other materials in vascular tissue engineering [266, 267].

To alleviate the limitation of low potential for vascular regeneration, Śmiga-Matuszowicz et al. used PLGA in combination with IS-based polyester, poly (isosorbide sebacate) (PISEB) to form an electrospun scaffold appropriate for vascular regeneration [268]. The obtained PLGA/PISEB scaffold exhibited appropriate biocompatibility, biodegradability, and high mechanical strength, while its biological characteristics were studied using HUVECs. The relative cell viability of HUVECs was approximately 90% (the threshold to distinguish biocompatible and cytotoxic materials was 70%), which once again proved that PLGA/PISEB is a biocompatible material [268, 269]. The genotyping of HUVECs collected after 48 h of culture on the surface of the PLGA/PISEB scaffold showed a potential pro-angiogenic expression profile and a specific anti-inflammatory effect [268, 270, 271]. In addition, after hydrolysis and degradation for up to 12 h, the PLGA/PISEB scaffold formed a highly developed structure, which may positively impact the stability of regenerated blood vessels [268, 272]. The combination of PLGA and natural gelatin through electrospinning enhanced the mechanical strength and biocompatibility of the scaffold, ha a strong antibacterial effect, enhanced the production of VEGF, and promoted angiogenesis [199, 273]. When used in combination with PCL, the resulting polymer scaffolds are better suited for vascular tissue engineering [274, 275]. When the three materials PLGA, PLLA and PLCL are used in combination, the proliferation of SMCs and ECs can be better promoted [200]. Wang et al. modified the obtained scaffold with heparin, which not only enhanced the anticoagulant effect of the vascular stent, but also accelerated the endothelialization of the modified vascular scaffold [201]. Atherosclerosis is the main reason for a variety of cardiovascular diseases and also a significant cause of morbidity and mortality in middle-aged and elderly people. Thus, the development of DDS for atherosclerosis is particularly urgent [276, 277]. Wu et al. designed an EGFP-EGF1 coupled PLGA nanoparticle DDS loaded with 3-(2-benzothiazolyl)-7-(diethylamino)coumarin (coumarin 6) and 1,1′-dioctadecyl-3,3,3′,3′-tetramethylindotricarbo cyanine iodide (DiR) and demonstrated that the drug could be effectively absorbed by targeted cells in atherosclerotic cell models in vitro and delivered to atherosclerotic plaque sites in vivo [121]. Admittedly, this study has some.

**Fig. 6:**
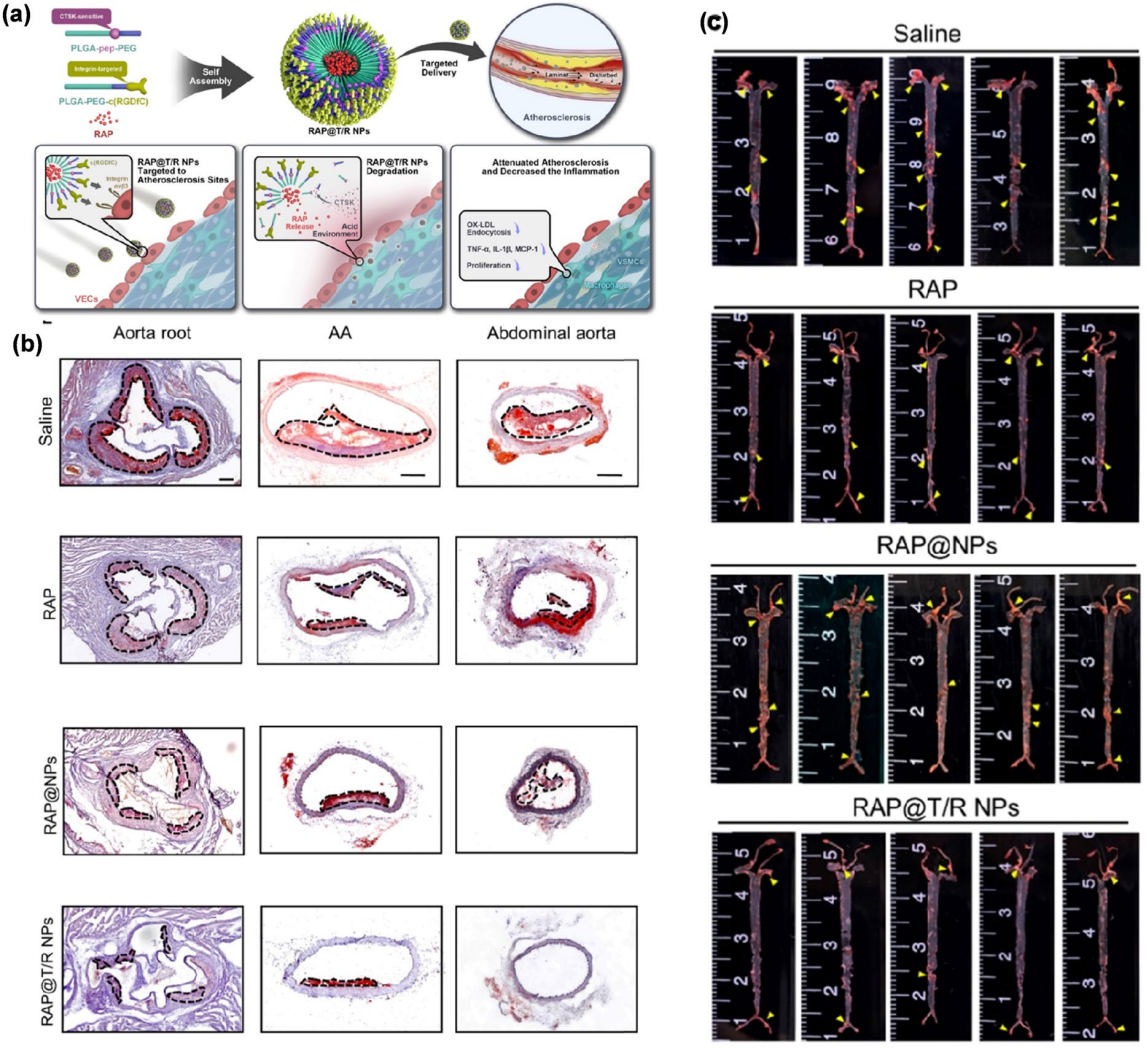
**a** Schematic diagram of the components of the RAP@T/R NPs and targeted delivery of rapamycin to treat atherosclerosis in response to CTSK. **b** ORO-stained cryosections of the aortic root, AA, and abdominal aorta (plaque lesions: dotted frame, Scale bar: 200 µm, n = 5). c**)** En face ORO-staining of aortas from ApoE.^−/−^ mice after treatment with different formulations (saline, rapamycin, RAP@NPs, RAP@T/R NPs at a dose of 0.5 mg/kg rapamycin twice a week, n = 5, plaque lesions: yellow arrowheads) (reprinted with permission from Ref. [204])

Inadequacies as it only explored the in vivo effect after 1 h of administration, which may be sufficient for diagnostic purposes. Thus, the information obtained from the study is insufficient for effective drug carriers for targeted therapy [121]. Further experiments verified that PEG-PLGA NPs can be used as drug delivery vehicles [278]. After this, Fang et al. also developed PEG-PLGA NP DDS loaded with rapamycin for selective delivery to the atherosclerotic site and improved in vivo safety (Fig. 6) [204]. When a PLGA scaffold is applied in vascular tissue engineering, it can be combined with a wide array of substances to achieve better results [202, 279].

## Summary and outlook

Currently, vascular injuries and diseases represent a significant threat to human health, and further development of vascular tissue engineering is of paramount importance for addressing the related clinical challenges. Polyesters such as PHA, PCL, PLA, and PLGA have been extensively employed in vascular tissue engineering due to their exceptional biocompatibility, optimal degradation time and rate, robust mechanical strength, and capacity to serve as a carrier for DDS. They can be employed as an alternative to vascular grafts, effectively regulating vascular repair and regeneration, preventing thrombosis, and reducing atherosclerosis. Furthermore, they can be employed in related therapeutic methods, such as vascular interventions. In the past 5 years, there has been rapid development of polyesters in vascular tissue engineering. Researchers have developed three-layer vascular grafts that can match the mechanical properties and elasticity of natural vascular tissue. These grafts are safe and non-toxic and can be used to target and deliver drugs to suitable sites, maintaining a specific concentration.

Nevertheless, it is evident that polyesters continue to exhibit certain limitations. Firstly, when polyesters are implanted into the human body as vascular grafts, they must remain in the body for several years or even decades, yet the long-term effects of their implantation remain unknown. A limitation that is evident in the current literature is the lack of long-lasting follow-up studies, which precludes an accurate assessment of the long-term effects. Consequently, further clinical studies, long-term observations, and follow-up studies are required to explore and validate the available results [280]. Secondly, the metabolic substances produced by the degradation of polyesters in the human body have the potential to alter the local tissue environment and even affect surrounding tissues, which may result in inflammation. This may necessitate the addition of other materials to the scaffolds. When PLGA is degraded, it generates acidic by-products, which can be effectively mitigated by the addition of magnesium hydroxide [281–283]. Thirdly, when employed in vascular tissue engineering, polyesters present certain limitations in terms of elasticity and mechanical strength. Although some stents have already demonstrated the capacity to match the mechanical properties of natural vessels, their mechanical strength remains inadequate. However, the performance of these scaffolds can be enhanced by employing multiple polyesters or alternative materials, such as the combination of PCL with PU, PCL with 1,4-diisocyanatobutane (BDI), and potentially by modifying the processing methods to achieve the desired mechanical strength [129, 284–286]. Fourthly, when utilising microspheres, nanoparticles and other carriers as drug delivery vehicles, it is essential to consider not only methods of increasing the quantity of drug transported, but also to identify strategies for minimising the long-term effects of these carriers on the human body. This is a requisite precautionary measure to reduce the incidence of adverse effects, the necessity of which is underscored by the need for further research. At present, the most commonly employed techniques include the incorporation of biologically active substances, enhancement of the porosity of the scaffolds, the introduction of additional biomaterials, and the development of appropriate processing methodologies.

In conclusion, polyesters are increasingly being employed in vascular tissue engineering, with notable current outcomes and considerable future potential. Nevertheless, further research and investigation are required to effectively address the health issues associated with vascular injuries and diseases. This article reviewed the distinctive properties, processing techniques, and structural characteristics of polyesters, providing a comprehensive overview of their specific applications in vascular tissue engineering from 2019 to 2024. Finally, we also outlined the limitations and future trends of polyesters in vascular tissue engineering.

## Data Availability

No datasets were generated or analysed during the current study.
